# 3D deep learning-based muscle volume quantification from thoracic CT as a surrogate for DXA-Derived appendicular muscle mass in older adults

**DOI:** 10.1007/s40520-025-03206-1

**Published:** 2025-10-21

**Authors:** Sabine Schluessel, Benedikt Mueller, Michael Drey, Olivia Tausendfreund, Michaela Rippl, Linda Deissler, Sebastian Martini, Ralf Schmidmaier, Sophia Stoecklein, Michael Ingrisch

**Affiliations:** 1 Department of Medicine IV, LMU University Hospital, LMU Munich, Munich, Germany; 2 Department of Radiology, LMU University Hospital, LMU Munich, Munich, Germany; 3 Munich Center for Machine Learning (MCML), Munich, Germany; 4 Konrad Zuse School of Excellence in Reliable AI, Munich, Germany

**Keywords:** Geriatric patient, Body composition, Machine learning, Muscle assessment, Computed tomography, Muscle fat infiltration

## Abstract

**Background:**

In order to identify patients with sarcopenia, the use of routine imaging could provide valuable support. One of the most common radiological examinations, especially in geriatric inpatient care, is CT thoracic imaging. Therefore, it would be desirable to generate muscle volumes from these images using automated body composition analysis. The aim of this study is to determine the muscle volumes of geriatric patients and to investigate to what extent these correspond to the values of one of the current reference standards in diagnosing sarcopenia, the Dual-energy X-ray Absorptiometry (DXA) measurement.

**Methods:**

This retrospective study included 208 geriatric patients (mean age: 81 ± 7 years, 53.4% women) treated at the Acute Geriatric Ward at LMU University Hospital between 2015 and 2022. All participants underwent DXA measurement to assess appendicular skeletal muscle mass (ASM). Pretrained deep learning models were used to analyze body composition from routinely obtained thoracic CT images. Correlations between CT and DXA data were calculated using Pearson correlations, taking into account different normalization variants (height^2^, weight, bone volume and total volume). Multivariable linear regression analysis was performed to predict DXA-measured ASM.

**Results:**

Women and men differed significantly in bone volume, muscle volume, and intramuscular fat. A reliable correlation was found between muscle volume from CT-thorax analysis and ASM from DXA, especially for absolute muscle volume (*r* = 0.669, *p* < 0.001) and muscle volume normalized to height^2^ (*r* = 0.529, *p* < 0.001). In regression analysis, CT muscle volume alone explained 44.5% of the variance in ASM (R² = 0.445, *p* < 0.001). When body weight was added, the model’s explanatory power increased significantly to 68.9% (R² = 0.689, *p* < 0.001). The fully adjusted model, which included height, age, and sex, further improved the explained variance only slightly (R² = 0.713, *p* < 0.001). Among all predictors, body weight showed the strongest effect, followed by CT muscle volume, while sex had no significant influence.

**Conclusion:**

The results show that the automated analysis of CT thoracic scans is a useful method for determining muscle volume and agrees well with the DXA analysis. Furthermore, the predictive value of CT muscle volume is significantly enhanced in combination with anthropometric parameters, particularly body weight. Further prospective studies are required to validate the findings and refine CT-based sarcopenia diagnostics.

## Background

One of the greatest challenges of the 21 st century is the aging population [1]. In this context, age-related diseases such as sarcopenia are becoming increasingly important [2]. Sarcopenia describes the loss of muscle strength and muscle mass, that limits independence, increases the risk of falls, and is associated with a higher mortality rate [3–5]. Therefore, it is crucial to develop simple and effective diagnostic methods for this condition [5].

Currently, several methods are available for diagnosing sarcopenia, including dual-energy X-ray absorptiometry (DXA), bioelectrical impedance analysis (BIA), magnetic resonance imaging (MRI) and computed tomography (CT) [2]. However, DXA and BIA are not universally accessible and require additional resources for both patients and healthcare systems [6, 7]. In contrast, using routinely obtained CT images to detect sarcopenia could be a more efficient alternative [8]. CT images are already routinely used for body composition analysis, muscle mass measurement, tumor diagnostics, and other conditions [8]. In particular, thoracic CTs are among the most common imaging procedures, with utilization in Europe increasing by more than 80% in recent years [9]. This availability makes them ideal for body composition analysis [8].

In the past, segmenting body structures in CT images was a very time-consuming manual process [10]. However, advances in artificial intelligence (AI) now enable automated approaches that significantly reduce analysis time [10]. Although AI offers great potential in research, it has not yet found a firm place in clinical practice. However, recent reviews emphasize that AI could be a promising tool for detecting sarcopenia in routine CTs [11]. Nevertheless, the current standard for diagnosing sarcopenia using CT is still based on analyzing the cross-sectional area of the thigh or lumbar muscles [2]. A weakness of the methods applied so far is the use of two-dimensional (2D) images. In most cases, the cross-sectional area of the muscles is measured at a single lumbar vertebra and normalized to height [8]. On this basis, specific thresholds for sarcopenia have been defined [8]. The literature pays particular attention to the third lumbar vertebra (L3), since the largest muscle cross-sectional area is found there [12, 13]. If L3 is not available, other lumbar or thoracic vertebrae can be used, although there is no agreement on which thoracic vertebra is best suited [8, 14]. However, studies show strong correlations between measurements at different thoracic levels, such as the fourth (T4) and the twelfth thoracic vertebra (T12) [15, 16]. A key problem, however, remains that the level of L3 is often not included in routine thoracic CTs. Therefore, there is an urgent need to use alternative approaches.

Although correlations between CT-derived muscle measurements and DXA-based assessments have been demonstrated [17] particularly for single-slice 2D measures at the L3 level —these approaches rely on abdominal imaging and are not always applicable in clinical settings.

Thoracic CTs, by contrast, are frequently acquired in routine care, especially among older adults. However, there is limited evidence on whether fully automated 3D volumetric muscle analysis from thoracic CT can provide a valid surrogate for appendicular muscle mass.

To address this gap, we investigated whether 3D muscle volumes extracted from routine thoracic CTs using a deep learning-based segmentation pipeline can accurately reflect DXA-measured appendicular muscle mass. This approach extends beyond traditional 2D slice-based metrics and may offer a more practical tool for muscle mass estimation.

Therefore, the primary objective of this study was to investigate whether muscle volumes derived from thoracic CT scans correlate well with DXA-derived appendicular skeletal muscle mass (ASM) in a geriatric cohort. ASM is a widely accepted reference standard for estimating whole-body muscle mass and diagnosing sarcopenia, and was therefore used as a comparator to assess whether thoracic CT-based muscle volumes can serve as a reliable surrogate measure. In addition, we aimed to determine which method of normalization (e.g., by height or body weight) yields the most accurate results. The secondary aim was to define diagnostic thresholds for sarcopenia based on muscle volumes obtained from the CT-thorax in order to develop a practical method for clinical applications in the future.

## Methods

### Patient characteristics

This retrospective study included individuals who were treated in the Acute Geriatrics Department of LMU Hospital between January 1, 2015, and August 31, 2022 (only hospitalized patients). Patients aged 65 years or older were included if they had undergone a thoracic CT scan as part of routine clinical care for an acute condition such as pneumonia, COPD exacerbation, tumor evaluation, or suspected pulmonary embolism. If an abdominal CT scan was also available, it was included for the analysis of muscle volumes at different lumbar vertebral levels; however, it was not mandatory for inclusion. In addition, they had to have undergone a whole-body DXA scan to assess muscle mass and their handgrip strength was measured. To ensure consistency, all CT scans, DXA scans and handgrip strength measurements were performed within a maximum period of 30 days. The study protocol was approved by the LMU Munich Ethics Committee (study no. 22–1057).

## Measurements

Height (cm) and weight (kg) were measured. Handgrip strength was assessed using a hydraulic handheld dynamometer (Jamar, Los Angeles, CA) with the patient seated upright and the arm positioned at a 90-degree angle of flexion. Three consecutive measurements were performed for each hand, and the highest recorded value was used for analysis.

## DXA

Body composition was measured by DXA (Lunar Prodigy, GE Healthcare Technologies, USA). The appendicular skeletal muscle mass (ASM) was obtained as the sum of appendicular lean mass of both arms and legs. ASM was then normalized by height squared (ASM/height^2^) [2].

## Body composition analysis

CT images of the thorax—and in a subgroup, also of the abdomen—acquired during routine clinical care were used for deep learning-based body composition analysis. Prior to the extraction of body composition markers, the CT images were resampled to a slice thickness of 5 mm to ensure a uniform dataset for further analysis. The body composition markers were then calculated using a fully automated evaluation pipeline. The pipeline and further details about the Smart Hospital Information Platform (SHIP)-AI software of the University Hospital Essen have been Published elsewhere in 2021 [18], and the pipeline has been applied in numerous publications over the past years [19–24]. All segmentations were checked visually, but no manual adaptions were necessary.

Our analysis focused primarily on the thorax, excluding the extremities. When available, data from abdominal CT scans were also included. The thoracic cavity was defined as the chamber enclosed by the rib cage, extending from the superior thoracic aperture to the diaphragm, and includes the trachea up to the level of the cricoid cartilage. The abdominal cavity was defined as the anatomical region extending from the diaphragm down to the symphysis pubis. For these areas, the volumes of the relevant body structures were determined. In alignment with our research objectives, the analysis specifically focused on muscle, bone, total fat, and intramuscular fat. All raw parameters were given as volumes (mL).

Additionally, as described by Salhöfer et al. and Keyl et al., we applied the myosteatosis index (%), determined by dividing the intramuscular adipose tissue volume (mL) by the total adipose tissue volume (mL) and multiplying the result by 100 [20, 22].

## Clinical definitions

According to the criteria of the European Working Group on Sarcopenia in Older People 2 (EWGSOP2), probable sarcopenia was defined as reduced handgrip strength- <27 kg for men and < 16 kg for women [2]. Confirmed sarcopenia was diagnosed when, in addition to low handgrip strength, the ASM/height^2^ measured by DXA was below 7.0 kg/m² for men and 5.5 kg/m² for women [2].

### Statistical analysis

For our statistical analyses, we used IBM^®^ SPSS^®^ Statistics version 29. Metric variables were reported as means with standard deviations (SD), while categorical variables were presented as frequencies and percentages. Group comparisons were performed using Student’s t-test for metric variables and the Chi-square test for categorical variables. When necessary, Fisher’s exact test was applied for categorical variables with small sample sizes.

To standardize muscle volume, adjustments were made for height squared, weight, bone volume, and total volume in both thoracic and abdominal CT scans.

The primary objective of this study was to compare muscle volumes derived from CT scans with the reference standard DXA measurements. To achieve this, we analyzed the correlation between CT-derived muscle volumes (and their normalization variants) and DXA-derived measures, including ASM and ASM/height², using Pearson’s correlation coefficient. Additionally, ASM and ASM/height² were visually plotted against muscle volume and its normalization variants.

In the final step, a standard multivariable linear regression analysis was conducted. The dependent variable was appendicular lean mass (in kg), while independent variables included thoracic muscle volume (in mL), body weight (kg), height (cm), age (years), and sex (0 = male, 1 = female). First, a univariate model was constructed using CT muscle volume as the sole predictor. A second model added body weight to assess the combined predictive value of anatomical and anthropometric data. Finally, a fully adjusted model included additional covariates: height, age, and sex. All variables were assessed for linearity (using scatterplots) and multicollinearity (Variance inflation factor > 5 presenting high multicollinearity) prior to inclusion. Standardized regression coefficients (β) were reported to assess the relative influence of each predictor. All predictors were entered simultaneously using the ENTER method in SPSS.

Cut-off points for CT-derived muscle volumes were calculated based on the established cut-off thresholds for ASM (< 15 kg for women, < 20 kg for men) and ASM/height² (< 5.5 kg/m² for women, < 7.0 kg/m² for men) as defined by the EWGSOP2 [2].

A p-value of < 0.05 was considered statistically significant throughout the analysis.

## Results

### Patient characteristics

A total of 208 patients (mean age: 81 ± 7 years; 111 women) met the inclusion criteria. Among all 208 patients, 57 (27.4%) were diagnosed with sarcopenia, with a higher prevalence observed in men. The average interval between the thoracic CT and the DXA scan was nine days. During the thoracic CT examination, 151 patients (72.6%) positioned both arms above the head. An abdominal CT scan was additionally available in a subgroup of 66 patients alongside the thoracic CT. A detailed overview of the patient characteristics is shown in Table 1. The results of the automated body composition analysis are summarized in Table 2. These show the mean and SD for the parameters muscle mass, bone mass, total body fat and intramuscular fat, separated by gender. For better comparability, the volumes of the different tissues in the thorax and abdomen were calculated without including the extremities. In the thoracic CT, the groups differed significantly in muscle volume, bone volume, and intramuscular fat volume, whereas in the abdominal CT, significant differences were observed only in muscle and bone volume.

**Table 1:** Study participants characteristics

Characteristics	All		Women			Men			*p*-value	
n (%)	208 (100.0)		111 (53.4)			97 (46.6)				
Age [years]	81 (7)		82 (8)			81 (7)			0.142^a^	
BMI [kg/m^2^]	24.9 (5.0)		24.5 (5.3)			25.4 (4.5)			0.199^a^	
Sarcopenia										
Handgrip strength [kg]	19.7 (8.5)		15.3 (4.9)			24.7 (8.9)			**< 0.001** ^a^	
ASM [kg]	17.5 (4.0)		15.6 (3.1)			19.6 (3.8)			**< 0.001** ^a^	
ASM/height^2^ [kg/m^2^]	6.3 (1.2)		6.0 (1.2)			6.7 (1.1)			**< 0.001** ^a^	
Presarcopenia (%)	116 (55.8)		58 (52.3)			58 (59.8)			0.275^a^	
Sarcopenia (%)	57 (27.4)		18 (16.2)			39 (40.2)			**< 0.001** ^a^	
Time intervals										
Handgrip strength & DXA [days]	6 (5)		7 (6)			6 (4)			0.222^a^	
Thoracic CT & DXA [days]	9 (6)		10 (6)			9 (6)			0.149^a^	
Abdominal CT & DXA [days]	10 (7)		11 (7)			9 (6)			0.316^b^	
Thoracic CT & handgrip strength [days]	11 (8)		11 (8)			10 (7)			0.376^a^	
Abdominal CT & handgrip strength [days]	11 (7)		12 (8)			10 (7)			0.437^b^	
CT imaging										
Positioning of both arms above the head (%)	151 (72.6)		76 (68.5)			75 (77.3)			0.202^a^	
Positioning of one arm above the head (%)	16 (7.7)		8 (7.2)			8 (8.2)				
Positioning both arms next to the body (%)	41 (19.7)		27 (24.3)			14 (14.4)				
Abdominal CT available (%)	66 (31.7)		32 (28.8)			34 (35.1)			0.336 ^a^	

^a^ Variable was available for 208 patients

^b^ Variable was available for 66 patients

ASM: appendicular skeletal muscle mass. BMI: body mass index. CT: computer tomography

P-values refer to comparisons between female and male participants

**Table 2:** Body composition analysis

Body tissue	All	Women	Men	*p*-value
n (%)	208 (100.0)	111 (53.4)	97 (46.6)	
Thoracic CT				
Muscle volume [L]	2.57 (0.70)	2.12 (0.40)	3.10 (0.61)	**< 0.001** ^**a**^
Bone volume [L]	1.82 (0.41)	1.52 (0.19)	2.18 (0.31)	**< 0.001** ^**a**^
Total fat volume [L]	5.17 (2.74)	5.02 (2.81)	5.34 (2.68)	0.413^a^
Intramuscular fat volume [L]	1.17 (0.58)	1.06 (0.53)	1.29 (0.62)	**0.004** ^**a**^
Myosteatosis index [%]	23.93 (5.65)	23.01 (6.24)	24.98 (4.69)	**0.012** ^**a**^
Abdominal CT				
Muscle volume [L]	5.13 (1.36)	4.31 (1.09)	5.61 (1.13)	**< 0.001** ^**b**^
Bone volume [L]	2.73 (0.58)	2.34 (0.43)	3.10 (0.44)	**< 0.001** ^**b**^
Total fat volume [L]	12.47 (8.17)	11.72 (7.14)	13.17 (9.08)	0.475^b^
Intramuscular fat volume [L]	1.66 (1.02)	1.53 (0.81)	1.78 (1.18)	0.311^b^
Myosteatosis index [%]	14.72 (4.48)	14.64 (4.54)	14.79 (4.47)	0.890^b^

^a^ Variable was available for 208 patients

^b^ Variable was available for 66 patients

CT: computer tomography

P-values refer to comparisons between female and male participants

### Correlation between CT- and DXA-Derived measurements

**Fig. 1:**
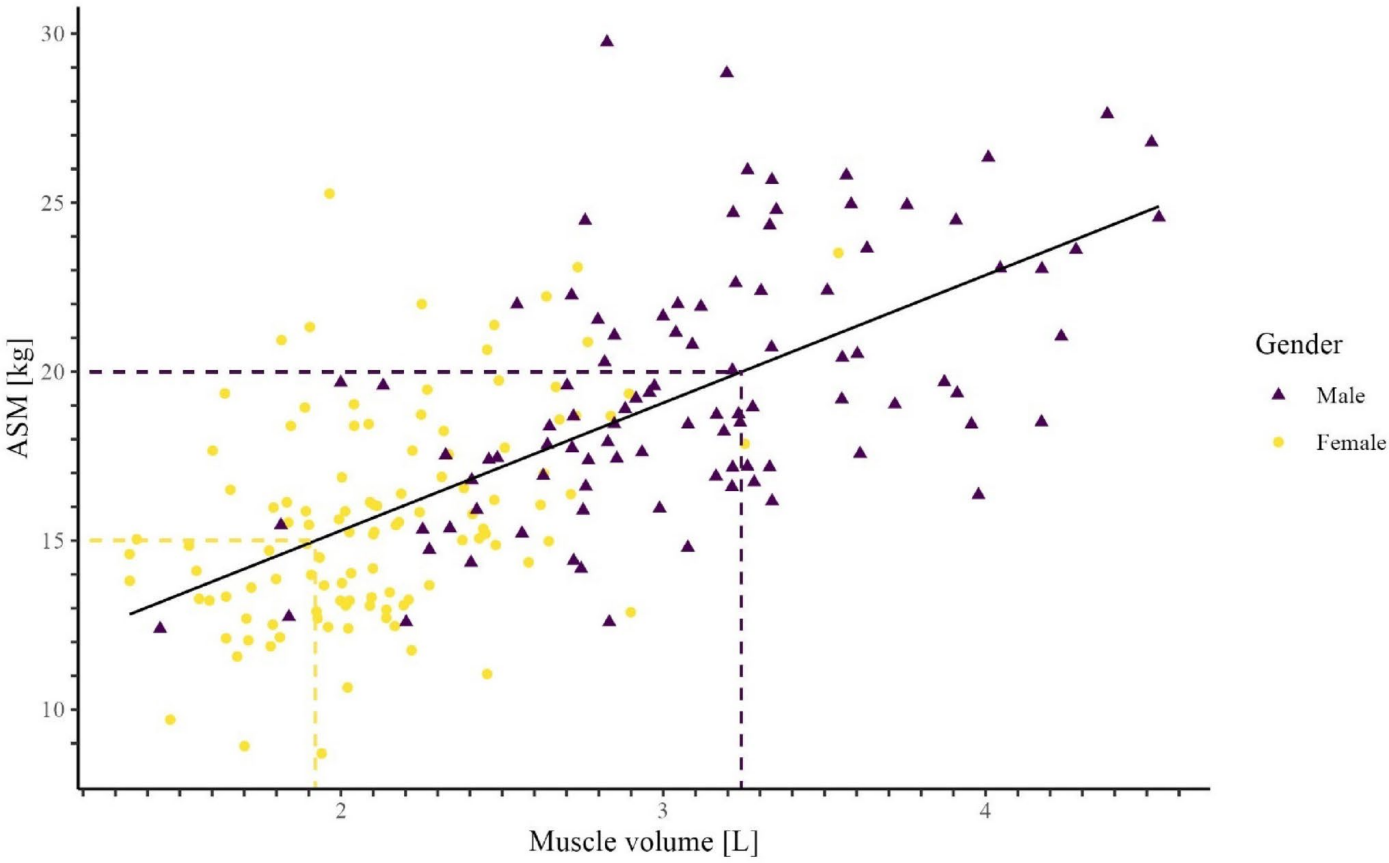
Scatterplot depicting the relationship between ASM (DXA) and muscle volume (CT-thorax). Abbrev: ASM: appendicular skeletal muscle mass. The colour scheme was specifically chosen so that it is easily distinguishable even for patients with red-green color deficiency. The dashed lines indicate the ASM thresholds according to the EWGSOP2 sarcopenia criteria

Table 3 shows the correlations between muscle volume and its normalizing variants (normalized for height², weight, bone volume, and total volume) from CT compared with ASM (or ASM/height²), derived from DXA. The highest correlation was observed between muscle volume from thoracic CT and ASM derived from DXA, with a Pearson correlation coefficient of 0.669 (*p* < 0.001), indicating a strong relationship. This specific correlation is graphically represented as a scatter plot in Fig. 1.

When examining individual lumbar vertebrae 3D slices, the strongest correlation was found at L2 (0.610, *p* < 0.001), although all levels from L1 to L5 demonstrated relatively similar correlation coefficients (Table 3).

**Table 3:** Correlation of ASM and ASM/height^2^ with muscle volume

		ASM		ASM/height^2^	
		Thoracic CT	Abdominal CT	Thoracic CT	Abdominal CT
Muscle volume	*Corr.*	**0.669** ^a^	**0.635** ^b^	**0.473** ^a^	**0.445** ^b^
	*p-value*	**< 0.001** ^a^	**< 0.001** ^b^	**< 0.001** ^a^	**< 0.001** ^b^
Muscle volume/height^2^	*Corr.*	**0.503** ^a^	**0.446** ^b^	**0.529** ^a^	**0.497** ^b^
	*p-value*	**< 0.001** ^a^	**< 0.001** ^b^	**< 0.001** ^a^	**< 0.001** ^b^
Muscle volume/weight	*Corr.*	0.064^a^	0.058^b^	−0.054^a^	−0.070^b^
	*p-value*	0.359^a^	0.642^b^	0.435^a^	0.574^b^
Muscle volume/bone volume	*Corr.*	**0.196** ^a^	0.121^b^	**0.252** ^a^	0.216^b^
	*p-value*	**0.004** ^a^	0.332^b^	**< 0.001** ^a^	0.081^b^
Muscle volume/total volume	*Corr.*	0.051^a^	−0.229^b^	0.014^a^	**−0.285** ^b^
	*p-value*	0.467^a^	0.064^b^	0.838^a^	**0.021** ^b^
Muscle volume L5	*Corr.*		**0.496** ^c^		**0.465** ^c^
	*p-value*		**< 0.001** ^c^		**< 0.001** ^c^
Muscle volume L4	*Corr.*		**0.553** ^c^		**0.414** ^c^
	*p-value*		**< 0.001** ^c^		**0.002** ^c^
Muscle volume L3	*Corr.*		**0.558** ^c^		**0.392** ^c^
	*p-value*		**< 0.001** ^c^		**0.004** ^c^
Muscle volume L2	*Corr.*		**0.610** ^c^		**0.475** ^c^
	*p-value*		**< 0.001** ^c^		**< 0.001** ^c^
Muscle volume L1	*Corr.*		**0.554** ^c^		**0.460** ^c^
	*p-value*		**< 0.001** ^c^		**< 0.001** ^c^

^a^ Variable was available for 208 patients

^b^ Variable was available for 66 patients

^c^ Variable was available for 52 patients

ASM: appendicular skeletal muscle mass. CT: computer tomography. Corr: pearson’s correlation

### Multivariable linear regression

A standard multiple linear regression analysis was conducted to assess the predictive value of CT-derived thoracic muscle volume for DXA-derived appendicular lean mass. In the univariate model, CT muscle volume alone explained 44.5% of the variance in appendicular lean mass (R² = 0.445, *p* < 0.001). When body weight was added to the model, the explained variance increased substantially to 68.9% (R² = 0.689, *p* < 0.001), indicating that weight is a major contributor to muscle mass prediction. A model with only weight reached an R² = 0.575, *p* < 0.001. A model with weight, age and height showed an R² = 0.646, *p* < 0.001. In the fully adjusted model, which included CT muscle volume, body weight, height, age, and sex, the explained variance increased slightly to 71.3% (R² = 0.713, adjusted R² = 0.706, *p* < 0.001). Among these predictors, body weight remained the strongest contributor (β = 0.531, 95% CI 0.122–0.170), followed by CT muscle volume (β = 0.318, 95% CI 0.001–0.002), while sex had no significant impact on DXA muscle mass (*p* = 0.986). The fully adjusted model is shown in Table 4.

**Table 4:** Multivariable linear regression predicting appendicular lean mass (DXA)

Predictor	B	SE	β (Beta)	*p*-value	95% CI for B
(Constant)	−13.393	3.898	−	< 0.001	[–21.079, − 5.708]
CT Muscle Volume (ml)	0.002	0.000	0.318	< 0.001	[0.001, 0.002]
Height (cm)	7.719	2.084	0.176	< 0.001	[3.609, 11.829]
Body weight (kg)	0.146	0.012	0.531	< 0.001	[0.122, 0.170]
Age (years)	0.042	0.021	0.078	0.042	[0.002, 0.083]
Sex (0 = male, 1 = female)	0.008	0.432	0.001	0.986	[–0.844, 0.860]

Model Summary: R² = 0.713, Adjusted R² = 0.706, F (5, 202) = 100.566, *p* < 0.001, Standard error of estimate = 2.16, *n* = 208

CT: computer tomography. B: unstandardized regression coefficient. SE: standard error. β (Beta): standardized regression coefficient. CI: confidence interval

### Cut-off points for CT-Derived muscle volumes

Table 5 provides cut-off values for muscle volumes in CT to identify low muscle mass, based on significant and positive correlations identified in Table 3. The cut-off values were determined by aligning them with the established thresholds for low muscle mass as defined by the EWGSOP2. Using the regression line, the corresponding muscle volume values for CT-derived body composition were calculated.

This process is illustrated in Fig. 1. For men, low muscle mass on DXA is defined as an ASM value below 20 kg (y-axis). According to the regression line, this corresponds to a muscle volume of 3.2 L, as shown on the x-axis. For women, the cut-off value for low muscle mass is an ASM below 15 kg, which, based on Fig. 1, corresponds to a CT-derived muscle volume cut-off of 1.9 L. The same approach was applied to determine the remaining cut-off scores *for* Table 5.

**Table 5:** Cut-off points for CT-Derived muscle volumes

	ASM				ASM/height^2^			
	Thoracic CT		Abdominal CT		Thoracic CT		Abdominal CT	
	Women	Men	Women	Men	Women	Men	Women	Men
Muscle volume [L]	1.921^a^	3.243^a^	3.649^b^	6.625^b^	1.543^a^	3.395^a^	2.971^b^	7.257^b^
Muscle volume/height^2^ [L/m²]	0.663^a^	1.207^a^	1.165^b^	2.516^b^	0.648^a^	1.160^a^	1.220^b^	2.439^b^
Muscle volume/bone volume	0.665^a^	2.189^a^			0.766^a^	1.938^a^		
Muscle volume L5 [L]			0.292^c^	0.792^c^			0.208^c^	0.697^c^
Muscle volume L4 [L]			0.245^c^	0.495^c^			0.214^c^	0.637^c^
Muscle volume L3 [L]			0.250^c^	0.500^c^			0.200^c^	0.635^c^
Muscle volume L2 [L]			0.268^c^	0.518^c^			0.217^c^	0.571^c^
Muscle volume L1 [L]			0.196^c^	0.446^c^			0.170^c^	0.514^c^

^a^ Variable was available for 208 patients

^b^ Variable was available for 66 patients

^c^ Variable was available for 52 patients

ASM: appendicular skeletal muscle mass. CT: computer tomography

## Discussion

A total of 208 patients (mean age: 81 ± 7 years; 53.4% women) were included in our study. Due to the strong correlations between CT-derived muscle volumes and DXA-derived muscle mass, we were able to demonstrate that muscle volume determination using thoracic CT scans is a valid method for determining muscle mass. In our cohort, the correlation coefficient derived from the thorax demonstrated even stronger correlations than the values obtained from L3. To the best of our knowledge, we are the first group to establish this approach in a geriatric cohort using DXA as reference standard.

### Patient characteristics and body composition

The baseline data reflect a typical geriatric patient population, with a mean age of 80 years and a normal weight BMI. About half of the patients met the EWGSOP2 criteria for presarcopenia, while about a quarter were classified as sarcopenic [2]. The higher prevalence of sarcopenia in men is consistent with previous research [25].

Body composition analyses also show sex-specific differences, particularly in muscle and bone volume, with men consistently showing greater volumes. One interesting parameter is intramuscular fat. Fat accumulation within the musculature is considered a typical characteristic of sarcopenia and the aging process [26]. In the CT thorax, men had a greater intramuscular fat volume (Table 2), which could be consistent with the higher number of sarcopenic patients in this group. Interestingly, however, this difference was not detected in the abdominal CT, which may reflect differences in muscle type, function, and disease context. Thoracic muscles are generally smaller, less active, and more susceptible to fat infiltration, especially in patients with chronic systemic diseases [27]. Additionally, the sample size for abdominal CT was smaller, so this observation should be interpreted with caution and confirmed in a larger cohort.

In the CT thorax, men had a higher myosteatosis index indicating higher intramuscular fat, which aligns with the greater number of sarcopenic patients in this group. Salhöfer et al. found that patients with idiopathic pulmonary fibrosis (mean age 70) who had a low sarcopenia index and a high myosteatosis index exhibited the shortest median survival time [20]. The myosteatosis index in our cohort (mean 23.9%, 25.0% in men) was even higher than the 19.5% reported by Salhöfer et al. [20]. This higher myosteatosis index is likely due to the fact that our patients represent a typical geriatric population, with a mean age approximately 10 years older than that of the cohort studied by Salhöfer et al. (81 years vs. 70 years) [20]. Since intramuscular fat infiltration naturally increases with age, it is plausible that our older patient group exhibits a higher degree of myosteatosis. Supporting this, Perkisas et al. found a myosteatosis index of 29.5% in a geriatric cohort of similar age, measured via CT scans of the thigh. In summary, our results appear reliable and typical for a geriatric population.

#### Correlation between CT- and DXA-Derived measurements

The central research question of our study was to evaluate the correlation between muscle volumes derived from CT images and appendicular skeletal muscle mass measured by DXA. Our data show a significant correlation between these two measurements (Table 3; Fig. 1). It should be noted that the DXA measurement primarily captures the muscle mass of the extremities, whereas in our study the muscle volume was determined in the thoracic CT. Therefore, complete agreement of the values was not to be expected. The strongest correlation (*r* = 0.669) was found between absolute muscle volume on CT and ASM, which further underlines the validity of this method for assessing sarcopenia. These results are consistent with previous studies, which indicate that thoracic CT can be used as a widely available imaging method to estimate muscle mass [20, 28]. The question remains which type of standardization is best suited?

According to the EWGSOP2 criteria, normalization to height is recommended for normal-weight patients, since taller people generally have a higher muscle mass [2]. Normalization to height squared was introduced for DXA measurement as early as 1998 by Baumgartner et al. and has since become established as the standard [29]. In our cohort, this method also showed a good correlation (Table 3), especially when both CT-based muscle volume and ASM were normalized to height^2^ (*r* = 0.529).

In addition, normalization to body weight was examined. This method has gained importance due to the consensus definition of European Society for Clinical Nutrition and Metabolism (ESPEN) and the European Association for the Study of Obesity (EASO) 2022, especially in the context of sarcopenic obesity, since pure volume measurement in obese patients can lead to an overestimation of muscle mass [30]. However, no significant correlation with this normalization was found in our cohort. One possible reason for this is the high proportion of normal-weight patients in our study. It is conceivable that this normalization would be more relevant in a cohort with a higher proportion of obese patients, highlighting the need for further investigation in future studies.

Furthermore, as suggested in other studies, muscle volume was correlated with bone volume, as this has proven to be a promising parameter in younger cohorts [20–22]. However, our study showed only a very small correlation, suggesting that this approach is less suitable for geriatric patients. One possible explanation is the high prevalence of osteopenia and osteoporosis in this age group, which could distort the relationship between muscle volume and bone density [31, 32].

Interestingly, the analysis of individual lumbar vertebrae revealed that muscle volume at the L2 level showed the strongest correlation with ASM in our cohort (*r* = 0.610). While previous studies primarily used the third lumbar vertebra (L3) as a reference for muscle mass determination, our results suggest that other vertebral heights may also be meaningful. Additionally, L3 did not exhibit the highest muscle volume in our cohort. The discrepancy compared to existing literature may be explained by the fact that we applied a 3D analysis of the lumbar vertebrae, whereas most previous studies relied on 2D axial slices. The choice of the optimal reference vertebra depends largely on the individual location of the maximum muscle cross-sectional area of a patient. This problem is avoided by the 3D analysis of muscle volume over a larger body segment using CT in this study, which allows a more accurate and independent assessment of muscle mass.

### Predictive value of CT muscle volume for DXA-Measured lean mass

CT-derived muscle volume was moderately associated with DXA-measured appendicular lean mass, explaining 44.5% of the variance when analyzed as a single predictor. However, when body weight was included, the explained variance increased markedly to 68.9%, underscoring the dominant role of body weight in determining lean mass. This aligns with existing literature, where body weight is often the most accessible surrogate for muscle quantity [2, 33, 34]. Interestingly, the addition of further covariates such as height, age, and sex led to only a marginal increase in R² (from 0.689 to 0.713; fully adjusted model Table 4). Among all predictors, body weight had the strongest standardized effect (β = 0.531), followed by CT muscle volume (β = 0.318). Importantly, sex did not contribute significantly to the prediction of DXA-measured lean mass once CT muscle volume and body weight were accounted for (*p* = 0.986). This does not imply an absence of sex differences in muscle mass per se—which are evident in the baseline characteristics—but rather reflects that sex does not add predictive value beyond body weight and CT-based muscle volume in the multivariable model. In summary, these results suggest that while CT-based muscle volume contributes independently to lean mass prediction, its utility is maximized in combination with body weight.

From a clinical perspective, the findings highlight the potential value of integrating CT metrics into multimodal muscle assessment, particularly in patients undergoing routine imaging.

#### Cut-Off points for CT-Derived muscle volumes

Using established EWGSOP2 thresholds for ASM, we derived cut-off points for muscle volumes from CT to identify low muscle mass (Table 5). These thresholds provide a practical framework for implementing CT-derived metrics in clinical practice. For example, an ASM of < 20 kg for men corresponds to a CT-derived thoracic total muscle volume of < 3.2 L, while an ASM of < 15 kg for women corresponds to a volume of < 1.9 L. The use of regression analysis to calculate these values ensures that they are closely aligned with existing diagnostic criteria for low muscle mass. Importantly, this approach allows the integration of muscle volume measurements from routine thoracic CT into sarcopenia diagnostics without requiring additional imaging or invasive procedures. While abdominal CT-derived values were also calculated, the limited availability of abdominal CT scans in our cohort (31.7%) suggests that thoracic CT is the more practical and widely applicable option for geriatric patients. This is in line with findings of a recent study indicating that body composition measurements derived from thoracic CT scans are more accurate than those based on the third lumbar vertebra [28].

#### Advantages and limitations of automated body composition analysis

There are some limitations to measuring muscle mass using DXA, which can be avoided by CT-based measurements. For example, the accuracy of DXA measurements is influenced by fluid retention, as occurs in heart, kidney or liver failure, which can lead to inaccurate results [8]. Furthermore, DXA does not allow differentiation between subcutaneous, visceral, and intramuscular fat, although intramuscular fat in particular is considered an important predictor of muscle quality in older patients [26, 35]. CT offers an advantage here, as it can determine not only muscle volume but also muscle quality based on density values (Hounsfield Units), which provides a more accurate assessment of myosteatosis [36]. Despite these advantages, CT is probably not suitable as a primary method for diagnosing sarcopenia due to the radiation exposure [37]. However, it offers significant added value in clinical contexts in which CT scans are routinely performed.

According to current guidelines, a diagnosis of sarcopenia always requires a combination of muscle mass and muscle function measurements [2]. Neither CT nor DXA can directly determine muscle function, which is why functional tests such as handgrip strength measurement or the chair-rising test remain indispensable [2].

#### Strengths and limitations of the study

One of the strengths of this study is the use of a large cohort of geriatric patients, ensuring that the findings are directly relevant to the population most affected by sarcopenia. Additionally, the use of automated AI-based body composition analysis reduces operator dependency and improves the reproducibility of measurements. However, there are limitations to consider.

Firstly, only 66 CT abdomen scans were recorded compared to 208 CT thoracic scans. Determining cut-off values from the CT abdomen was of secondary importance, as numerous 2D cut-off values for body composition are already available from the CT abdomen and the focus of this study was on CT chest imaging. Nevertheless, it should be taken into account that sarcopenia is a systemic condition. Since appendicular muscle mass and core strength are more directly linked to physical function and fall risk, our findings may not fully reflect the broader musculoskeletal status of the patients. Therefore, caution is warranted when generalizing these results to overall sarcopenic burden or functional capacity.Second, the time interval between CT imaging and DXA measurements could not be further reduced due to the retrospective nature of the study. While comparable studies allowed a maximum interval of 15 days between examinations, our study extended this to 30 days. However, the average interval was only 10 days, which aligns with the typical length of a hospital stay [24, 28].

Third, formal statistical testing to compare the diagnostic performance of the proposed CT-based parameter with existing measures—such as likelihood ratio tests for nested models—was not performed, as the primary objective of this study was to demonstrate proof of concept. A more detailed statistical evaluation, including confidence intervals (e.g., derived via bootstrapping) and comparative model performance metrics, is planned in future studies using larger, prospectively collected datasets.Finally, the retrospective study design meant that the arm position of patients during the CT examination was not standardized, as shown in the baseline table. To ensure consistency, the extremities were excluded from the CT muscle mass analysis. Although the majority of patients (72.6%) had both arms positioned cranially above the head during CT acquisition, varying arm positions may have influenced muscle stretching and, consequently, the measured muscle volumes in the thoracic region. Given the retrospective and routine nature of the imaging, no standardized positioning protocol was applied.

Therefore, in larger cohorts, future research should aim to systematically assess whether arm positioning has a relevant impact on thoracic muscle volume measurements and whether including the muscle mass of the visualized upper extremities provides additional predictive value for clinical endpoints.

Such analyses could help to clarify the extent to which variations in patient positioning affect the reliability of CT-based muscle assessments in routine clinical practice.

## Conclusion

Our findings support the integration of CT-based muscle volume analysis into routine clinical workflows for diagnosing sarcopenia, particularly in settings where DXA is not readily available. CT muscle volume alone showed a moderate correlation with DXA-derived muscle mass, which was significantly enhanced when combined with body weight. Future research should aim to validate these cut-off values in larger, prospective cohorts and explore the impact of including additional factors, such as muscle quality and fat infiltration, into diagnostic algorithms.

## Data Availability

No datasets were generated or analysed during the current study.
